# Detection and quantification of viable but non-culturable *Legionella pneumophila* from water samples using flow cytometry-cell sorting and quantitative PCR

**DOI:** 10.3389/fmicb.2023.1094877

**Published:** 2023-01-30

**Authors:** Muhammad Atif Nisar, Kirstin E. Ross, Melissa H. Brown, Richard Bentham, Giles Best, Harriet Whiley

**Affiliations:** ^1^College of Science and Engineering, Flinders University, Bedford Park, SA, Australia; ^2^College of Medicine and Public Health, Flinders University, Bedford Park, SA, Australia; ^3^Flow Cytometry Facility, Flinders University, Bedford Park, SA, Australia

**Keywords:** Legionnaires’ disease, premise plumbing, engineered water system, potable water, ISO11731:2017-05, ISO/TS12869:2019

## Abstract

*Legionella pneumophila* is a waterborne pathogen and, as the causative agent of Legionnaires’ disease, a significant public health concern. Exposure to environmental stresses, and disinfection treatments, promotes the formation of resistant and potentially infectious viable but non-culturable (VBNC) *Legionella*. The management of engineered water systems to prevent Legionnaires’ disease is hindered by the presence of VBNC *Legionella* that cannot be detected using the standard culture (ISO11731:2017-05) and quantitative polymerase reaction (ISO/TS12869:2019) methods. This study describes a novel method to quantify VBNC *Legionella* from environmental water samples using a “viability based flow cytometry-cell sorting and qPCR” (VFC + qPCR) assay. This protocol was then validated by quantifying the VBNC *Legionella* genomic load from hospital water samples. The VBNC cells were unable to be cultured on Buffered Charcoal Yeast Extract (BCYE) agar; however, their viability was confirmed through their ATP activity and ability to infect amoeba hosts. Subsequently, an assessment of the ISO11731:2017-05 pre-treatment procedure demonstrated that acid or heat treatment cause underestimation of alive *Legionella* population. Our results showed that these pre-treatment procedures induce culturable cells to enter a VBNC state. This may explain the observed insensitivity and lack of reproducibility often observed with the *Legionella* culture method. This study represents the first time that flow cytometry-cell sorting in conjunction with a qPCR assay has been used as a rapid and direct method to quantify VBNC *Legionella* from environmental sources. This will significantly improve future research evaluating *Legionella* risk management approaches for the control of Legionnaires’ disease.

## 1. Introduction

*Legionella* is an opportunistic premise plumbing pathogen associated with Legionnaires’ disease (LD) and Pontiac fever. It is ubiquitously present in natural and engineered water systems (Fields et al., 2002). Aerosols generated from cooling towers, showers, humidifiers and other fabricated water distribution systems are major sources of LD (Bartram et al., 2007). Within these engineered water sources, microbial biofilms and host protozoa protect *Legionella* from environmental stresses and disinfection procedures. Furthermore, disinfection protocols (Allegra et al., 2008; Casini et al., 2018) and environmental stresses (Li et al., 2014) cause culturable *Legionella* to enter a viable but non-culturable (VBNC) state. VBNC *Legionella* tolerate environmental stresses and are still able to infect human macrophages (Dietersdorfer et al., 2018) and alveolar epithelial cells (Epalle et al., 2015). Under favorable conditions, VBNC *Legionella* can be resuscitated using protozoan hosts to generate culturable *Legionella* (Steinert et al., 1997; Garcia et al., 2007).

Over the last decade, in the United States and across Europe the number of reported LD cases has been significantly increasing (The European Centre for Disease Prevention and Control, n.d.; Smith et al., 2007; National Notifiable Diseases Surveillance System and Centers for Disease Control and Prevention, 2018). In 2022, the European Centre for Disease Prevention and Control reported 8,372 confirmed cases of LD, of which 66.9% were community associated and 5.1% were nosocomial infections (The European Centre for Disease Prevention and Control, n.d.). During COVID-19 pandemic, lockdown resulted in water stagnation in some buildings, which ultimately increased *Legionella pneumophila* contamination (Liang et al., 2021; Chao and Lai, 2022). Furthermore, climate change, urbanization and new energy conservation approaches are also increasing the risk of legionellosis (both LD and Pontiac fever; Connolly et al., 2021; Gattuso et al., 2022). As such there is a need for improved risk management protocols to reduce the risk of legionellosis.

One of the challenges preventing the improved control of *Legionella* in engineered water systems is the uncertainty associated with standard detection methods (Whiley, 2016). The International Organization for Standardization (ISO) provides two protocols for the detection and quantification of *Legionella* contamination in potable water. ISO11731:2017-05 is a culture based method that detects only culturable *Legionella* (International Organization for Standardization, 2017); whereas, ISO/TS12869:2019 is a quantitative PCR (qPCR) based assay which estimates the bacterial genomic load (International Organization for Standardization, 2019). The culture method is considered the gold standard for *Legionella* detection and characterization. However, it is time consuming, taking 10–14 days, and does not detect VBNC *Legionella* resulting in underestimation of *Legionella* numbers and false negatives (Whiley and Taylor, 2016; International Organization for Standardization, 2017). In contrast, the qPCR method is faster (International Organization for Standardization, 2019); however, it quantifies both the live, VBNC and dead *Legionella*, resulting in overestimations and false positive results (Whiley and Taylor, 2016).

The concentration of culturable *Legionella* is typically low in potable water, however, previous studies have found high concentration of *Legionella* DNA using qPCR quantification (e.g., 10^3^–10^7^ GU/L) in culture negative potable water samples (Casini et al., 2018; Dai et al., 2019; Hayes-Phillips et al., 2019). Several methods have been used to try to overcome this discrepancy observed between the two standard methods (culture and qPCR). Ethidium monoazide (EMA) or propidium monoazide (PMA) based viability qPCR (Delgado-Viscogliosi et al., 2009; Taylor et al., 2014; Zacharias et al., 2015) and fluorescence *in situ* hybridization (FISH; Whiley et al., 2011) assays are alternative approaches designed to differentiate between viable and non-viable *Legionella* from environmental samples. However, background microbial populations compromise the validity of both techniques (Zacharias et al., 2015) and PMA and EMA have a concentration depended cytotoxic effect that can make it challenging to use in environmental samples of with unknown cell concentrations (Fittipaldi et al., 2012). Also, VBNC bacterial cells show low metabolic activity and rRNA contents, as such, FISH is not an effective technique to detect and characterize VBNC *Legionella* (Satoh et al., 2002). Catalyzed reporter deposition FISH (CARD-FISH) technique was designed to overcome lower contents of rRNA. It was used for direct enumeration of *L. pneumophila* from hospital water system (Kirschner et al., 2012). However, CARD-FISH detect and enumerate non-viable cells as well. Alternatively, combined microcolonies cultivation-FISH-solid phase cytometry assay was designed to detect and quantify viable *Legionella* (Baudart et al., 2015). However, this method cannot detect VBNC *Legionella*. *Legionella*-amoebae coculture assay is another technique used to detect VBNC *Legionella* from environmental samples (Conza et al., 2013). However, it is very time-consuming and is unable to quantify the population of VBNC *Legionella*.

Flow cytometric analysis of environmental samples has previously been used to characterize total bacterial VBNC populations (Sachidanandham et al., 2005; Khan et al., 2010). However, this method does not enable the discrimination and quantification of specific species from mixed bacterial population (Zacharias et al., 2015). Previous studies have also used flow cytometry to characterize VBNC *Legionella* generated by physical and chemical disinfection procedures, but these studies used pure cultures of *L. pneumophila* (Allegra et al., 2008; Mustapha et al., 2015). Another method used flow cytometry with specific fluorogenic antibodies to detect *L. pneumophila* from environmental samples (2010). In this method, FITC (ab20818, abcam) and Alexa (GTX40943, GeneTex) conjugated *L. pneumophila* sg1 (ATCC^®^ 33152^™^) specific polyclonal antibodies were applied to detected *L. pneumophila* sg1 (ATCC^®^ 33152^™^) which had been used to spike a water sample. Antibodies required for a universal assay must target epitope(s) that are well expressed in all serogroups and VBNC *Legionella*. In our study, to overcome this problem, instead of bacteria specific antibodies, dual staining flow cytometry-cell sorting was used, and genomic load estimated by a standard qPCR assay.

The aim of this study was to develop a rapid and robust “viability based flow cytometry-cell sorting and qPCR” (VFC + qPCR) assay to detect and quantify VBNC *Legionella* from engineered water systems. Following the optimization of the assay using pure *Legionella* cultures, its performance was validated using environmental water samples. It was also used to assess the impact of the pre-treatments described in the ISO11731:2017-05 culture method on *Legionella* recovery.

## 2. Materials and methods

### 2.1. Microbial strains, culture media, and growth conditions

*Legionella pneumophila* subsp. *pneumophila* Philadelphia serogroup 1 (ATCC^®^ 33152^™^) was cultured on BCYE (buffered charcoal yeast extract: CM0655, Oxoid Ltd.) agar supplemented with GVPC (glycine, vancomycin, polymyxin B and cycloheximide: SR0152, Oxoid Ltd.) and *Legionella* growth supplement (buffer/potassium hydroxide, ferric pyrophosphate, _L_-cysteine and α-ketoglutarate: SR0110C, Oxoid Ltd.); and incubated at 37 ± 1°C for 3–4 days. *Escherichia coli* HS (pF*amp*) R (ATCC^®^ 700891^™^) and *Acinetobacter calcoaceticus* (environmental isolate) were cultured on nutrient and MacConkey agar, respectively; and incubated at 37 ± 1°C for 24 h. *Acanthamoeba polyphaga* (ATCC^®^ 30461^™^) cells were cultured in a T25 flask (156367, Nunc^™^ EasYFlask^™^, Thermo Fisher Scientific) containing 4.5 mL PYG broth (2% peptone, 0.2% yeast extract and 1.8% _D_-glucose) supplemented with 10% heat inactivated fetal bovine serum (FBS: 10100139, Gibco^®^, Thermo Fisher Scientific) and incubated at 25 ± 1°C for 4–5 days. Whenever washing was required 1X phosphate-buffered saline (PBS: 003002, Invitrogen^™^, Thermo Fisher Scientific) and 1X Page’s saline (0.12 g NaCl, 0.004 g MgSO_4_.5H_2_O, 0.004 g CaCl_2_.2H_2_O, 0.142 g Na_2_HPO_4_, and 0.136 g KH_2_PO_4_ per liter distilled water, pH 6.8 ± 0.2) were used for bacterial strains and amoebae, respectively.

### 2.2. Determining the impact of sample processing according to ISO11731:2017-05 on *Legionella* culturability

A water sample spiked with 10^6^ CFU/L *L. pneumophila* was prepared. The sample was concentrated through filtration. After filtration, the contents present on the membrane were carefully resuspended in 1X PBS. This concentrated sample was processed according to the ISO11731:2017-05 standard culture method, which recommends heat or acid pretreatment to remove background microbes from water samples. For the heat treated sample, 2 mL of sample was heated at 50 ± 1°C for 30 ± 2 min, whereas for the acid pretreatment procedure, 1 mL sample was mixed with 9 mL of acid buffer (3.5 mL 0.2 M HCl and 25 mL 0.2 M KOH, final pH 2.2) and incubated at room temperature for 5 ± 0.5 min (International Organization for Standardization, 2017). Then untreated, heat and acid treated samples were cultured on BCYE-GVPC agar and tested using the VFC + qPCR assay described below. The data was analyzed using R (version 4.2.2) package agricolae (version 1.3-5; De Mendiburu, 2021). Firstly, normality was assessed using the Shapiro–Wilk test, then one way ANOVA (analysis of variance) was performed followed by Tukey’s HSD (honestly significant difference) test. Finally, graph was designed using ggplot2 (version 3.3.6) in the R environment (Wickham, 2016).

### 2.3. Viability based flow cytometry-cell sorting and qPCR assay (VFC + qPCR) development

#### 2.3.1. Preparation of *Legionella* suspension

To prepare *Legionella* suspension, 5 mL of 1X PBS (temperature preadjusted to 37 ± 1°C) was pipetted onto an agar plate containing *Legionella* colonies. These colonies were harvested by gently scrapping with a spreader to resuspended them in the PBS. The suspension containing harvested cells was then pipetted into a sterile 10 ml tube and repeatedly pipette mixed. Finally, 2.5 mL of the cell suspension was transferred to a 10 mL sterile tube containing 2.5 mL of PBS and again pipette mixed thoroughly. This was used as bacterial stock solution and diluted accordingly.

#### 2.3.2. Flow cytometry-cell sorting assay

The BD^™^ cell viability kit (349480, BD^™^) was used for staining and estimation of alive, dead and injured cell populations (Alsharif and Godfrey, 2002). Briefly, 100 μL of *Legionella* cells were mixed in 400 μL filter sterilized staining buffer (1 mM EDTA and 0.01% tween-20 in 1X PBS, pH 7.4 ± 0.1). In this mixture 420 nM of thiazole orange (TO; λ_(excitation)_/λ_(emission)_: 512/533 nm) and 48 μM propidium iodide (PI; λ_(excitation)_/λ_(emission)_: 537/618 nm) were added and vortexed. Sample tubes were incubated at 5°C for 15 min, then 50 μL of counting beads were added in each tube. Using a BD^™^ FACSAria^™^ Fusion instrument (BD Biosciences), cells were analyzed and the TO (FL1) vs. PI (FL3) fluorescence plots were used to distinguish alive, dead and injured cells. Untreated cells and heat killed cell (75°C for 10 min) were used as controls to define gates distinguishing alive and dead cell populations, respectively. The bacterial populations were first gated based on their forward (FSC) and side scatter (SSC) properties (Supplementary Figure S2). During optimization of the protocol, control samples containing predominantly dead or alive *Legionella* cells were used to position the quadrant gate. A blank control of buffer containing dyes was also included to confirm that debris was not being stained with the dyes and was not included in the gated populations (Supplementary Figure S1). All gated cell populations were examined to monitor viability and culturability of *Legionella*. Depending upon samples type and cell number, approximately 10^3^–10^6^ alive, dead, and injured cell fractions were then sorted into different tubes for further characterization.

#### 2.3.3. Interference of viability dyes in quantitative PCR

To confirm that TO and PI dyes did not interfere with the qPCR assay, sorted cell fractions (10^3^–10^5^ cells from each fraction) were subjected to DNA extraction and qPCR. The results of TO and PI dyed cell fractions were compared with the unstained *Legionella* and gBlocks gene fragments (IDT^™^). DNA was extracted using the Aquadien^™^ DNA extraction and purification kit (3578121, BIO-RAD Laboratories Ltd.). According to the guidelines of ISO/TS12869:2019 (International Organization for Standardization, 2019), the 16S rDNA gene was amplified and quantified. Briefly, gBlocks gene fragments (IDT™) were used as standard DNA with 10-fold serial dilution with concentrations ranging from 100 pg/μL to 0.00001 pg/μL. Using 2X SsoAdvanced^™^ universal probes supermix (172–5281, BIO-RAD Laboratories Ltd.) and 16S rDNA specific oligos (BIO-RAD Laboratories Ltd., Table 1), the reaction mix was prepared and subjected to a Rotor-Gene Q thermal cycler (QIAGEN Ltd.) for qPCR assay. In the assays, a channel with λ_(source)_ 470 nm and λ_(detector)_ 510 nm was used for detection of 6-carboxyfluorescein (λ_(excitation)_/λ_(emission)_ 495/520 nm) and Iowa Black^®^ FQ quencher labeled fluorogenic probe (BIO-RAD Laboratories Ltd.). Based on 10-fold serial dilution and qPCR assay, the limit of detection and limit of quantification were estimated. A standard curve was used to estimate the *Legionella* GU/L from alive, dead and injured cell fractions. All qPCR assays were performed in triplicate and mean C_T_ values were used for all calculations.

**Table 1:** Sequence of oligos and fluorogenic probes.

Name	Sequence/Fluorogenic signal (5′ → 3′)	References
*Legionella* PCR primers and probe		
Forward primer	GGAGGGTTGATAGGTTAAGAGCT	International Organization for Standardization (2019)
Reverse Primer	CCAACAGCTAGTTGACATCGTTT	
Probe	FAM-AGTGGCGAAGGCGGCTACCT-BHQ1	
Fluorescence *in situ* hybridization probes		
LEG705	Alexa Fluor 488-CTGGTGTTCCTTCCGATC	Manz et al. (1995)
EUK1209	Alexa Fluor 647-GGGCATCACAGACCTG	Lim et al. (1993)

### 2.4. Assay validation using *Legionella* pure cultures

#### 2.4.1. Thermal treatment

To study thermal tolerance, *L. pneumophila* was resuspended in 1X PBS at ≈ 10^8^ CFU/mL (λ_600nm_ 0.2) and heat treated at 37 to 75°C for 10 min, with gentle shaking after regular intervals of 3 min. To investigate effect of exposure time, cells were heated at 55°C in 1 min increments from 0 to 10 min. The time required to attain desired temperature within the bacterial vials was also considered. Following the thermal treatment vials were placed at 5°C for 60 min. All treated cells were analyzed by flow cytometry and sorted into alive, dead and injured (suspected VBNC) cell fractions (as described in section Flow cytometry-cell sorting assay).

#### 2.4.2. Culturability of VBNC *Legionella*

Culturability of sorted cell fractions was determined using BCYE agar (CM0655, Oxoid Ltd.) containing *Legionella* growth supplement (SR0110C, Oxoid Ltd.) and *Legionella* enrichment broth (M1399-100G, Himedia^®^) containing *Legionella* growth supplement (FD016A-5VL, Himedia^®^). For culturing, from 100 to 300 sorted cells from each fraction was inoculated onto each growth medium and incubated at 37 ± 1°C for 5–7 days.

#### 2.4.3. Viability of VBNC *Legionella*

The viability of non-culturable cells was determined using two methods: an ATP estimation assay and an amoebae coculture assay.

##### 2.4.3.1. ATP estimation assay

ATP contents were estimated using a luciferase-based ATP determination kit (A22066, Thermo Fisher scientific) according to the manufacturer’s instructions. The quantity of ATP in sorted cell fractions was estimated as a function of luminescence measured at 540 nm using a CLARIOstar^®^ microplate reader (BMG Labtech GmbH).

##### 2.4.3.2. Amoebae coculture assay

In this assay, 100 μL (10^7^ *Legionella*) of each of the sorted fractions, or a filter concentrated environmental water sample, were inoculated on 10^6^ *A. polyphaga* cells growing in infection medium (FBS-PYG broth and Page’s saline in 1: 10 ratio) and incubated at 25 ± 1°C for 7 days (multiplicity of infection of 10; Moffat and Tompkins, 1992). Internalization and resuscitation of *Legionella* within amoeba hosts was confirmed through FISH using Alexa Fluor 488 labeled *Legionella* LEG705 and Alexa Fluor 647 labeled eukaryotic universal EUK1209 fluorogenic probes (Invitrogen^™^, Table 1; Amann et al., 1990; Manz et al., 1995; Whiley et al., 2011). Briefly, infected cells resuspended in 1X PBS were fixed with 4% paraformaldehyde. Fixed cells were placed on a slide, air dried and dehydrated in an ethanol series: 50% ethanol/2 min, 80% ethanol/2 min and 96% ethanol/2 min. Fixed, and dehydrated cells were covered with 200 μL hybridization buffer (0.9 M NaCl, 0.01% SDS, 20 mM Tris–HCl pH 7.6) containing 100 ng of each probe and placed in a dark humidified chamber at 55 ± 1°C for 100 min. To remove unbound probes, cells were washed with warm wash buffer (0.9 M NaCl, 0.01% SDS, 20 mM Tris–HCl pH 7.6). Finally, the slide was washed with Milli-Q^®^ water, air dried, mounted with 20 μL of CitiFluor^™^ AF1 (17970–25, Electron Microscopy Sciences) and examined under fluorescence microscope (AX70, Olympus). Nonspecific binding of these probes was tested on *E. coli*.

### 2.5. Assay validation using mixed microbial samples

Four water samples spiked with different concentrations of *L. pneumophila*, *E. coli*, *A. calcoaceticus* and *A. polyphaga* were prepared (Table 2). All water samples were heated at 50°C for 15 min, then vacuum filtered onto a 47 mm diameter 0.2 μm polycarbonate membrane (GTTP04700, Isopore^™^). The filter membrane was transferred into a sterile tube containing 1,000 μL 1X PBS and vortexed. Presence of culturable *Legionella* in all four samples were confirmed using standard culturing method (International Organization for Standardization, 2017). From each resuspended sample, 200 μL was mixed in 300 μL filter sterilized staining buffer and used for flow cytometry-cell sorting. The sorted VBNC fractions were subjected to qPCR to estimate *Legionella* GU/L (as described in section Interference of viability dyes in quantitative PCR).

**Table 2:** Mixed microbial water samples spiked with *Legionella pneumophila*.

Name	Microorganism	Concentration/L
Sample 1	*Legionella pneumophila*	≈4.5 × 10^6^ CFU
Sample 2	*Legionella pneumophila*	≈4.5 × 10^6^ CFU	*Acanthamoeba polyphaga*	≈4.5 × 10^6^ Cells
Sample 3	*Legionella pneumophila*	≈4.5 × 10^6^ CFU	*Acinetobacter calcoaceticus*	≈9 × 10^7^ CFU	*Escherichia coli*	≈9 × 10^7^ CFU
Sample 4	*Legionella pneumophila*	≈4.5 × 10^6^ CFU	*Acinetobacter calcoaceticus*	≈9 × 10^7^ CFU	*Escherichia coli*	≈9 × 10^7^ CFU	*Acanthamoeba polyphaga*	≈4.5 × 10^6^ Cells

### 2.6. Assay validation using environmental water samples

From an Australian hospital, shower water (1.15 L) samples were collected in sterile screw capped wide-mouth plastic bottle. Culturable *Legionella* was detected using Legiolert kit (IDEXX) and standard culture method (International Organization for Standardization, 2017). For the standard culture method, 1 L water sample was concentrated through filtration. The concentrated residues were resuspended in 5 mL distilled water and subjected to both acid and heat pre-treatment steps. The treated sample was plated on BCYE-GVPC agar and incubated under standard culturing conditions (as described in section Microbial strains, culture media and growth conditions). For the Legiolert kit method, 100 mL sample water was processed according to the manufacturer’s protocols. Using the Aquadien™ DNA extraction and purification kit DNA was extracted from the water sample and *Legionella* GU/L was estimated. To detect presence of any VBNC *Legionella*, the PCR positive and culture negative samples were subjected to amoeba coculture assay. Finally, these *Legionella* PCR positive and culture negative shower water samples were processed to detect and quantify VBNC *Legionella* as described in section Flow cytometry-cell sorting and qPCR assay development.

## 3. Results

### 3.1. ISO sample processing protocol and *Legionella* culturability

**Figure 1:**
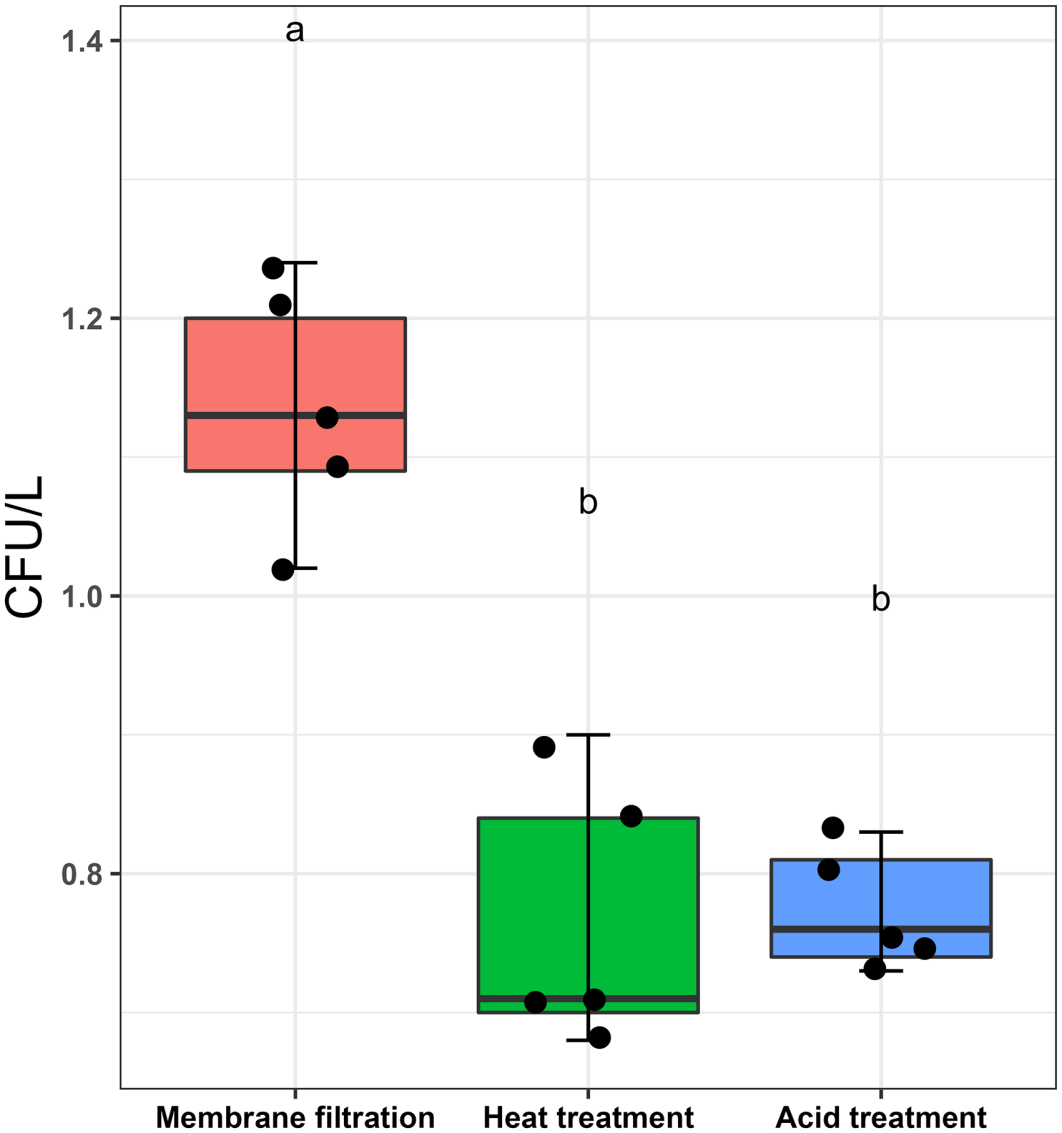
Effect of sampling processing methods (ISO11731:2017-05) on *L. pneumophila* culturability. *L. pneumophila* spiked water samples were membrane filtered (no pre-treatment used as control), followed by heat (50°C/30 min) and acid (KCl-HCl with pH 2.2/5 min) pre-treatment method. These pretreatment methods transformed ≈30% culturable *Legionella* into a non-culturable state. Data are the mean with standard deviation of five replicates, while the same letter within the same treatment is statistically similar according to Tukey’s HSD test (*p* < 0.001).

*L. pneumophila* spiked water samples were assayed according to ISO11731:2017-05 (International Organization for Standardization, 2017). Figure 1 shows that the highest culturable *Legionella* numbers were obtained by directly inoculating the sample after membrane filtration (1.136 ± 0.087 CFU/L). There were statistically significant differences between the control sample (no pre-treatment) and the two pre-treatments (*p* < 0.001). Both acid and heat decontamination steps transformed culturable *Legionella* to non-culturable cells and decreased the cell numbers to 0.774 ± 0.043 CFU/L and 0.766 ± 0.097 CFU/L, respectively. These results demonstrate that both sample pre-treatment procedures also effect the reliability of the ISO culturing assay (ISO11731:2017-05).

### 3.2. Assay optimization

#### 3.2.1. Flow cytometry and differentiation of cells

**Figure 2:**
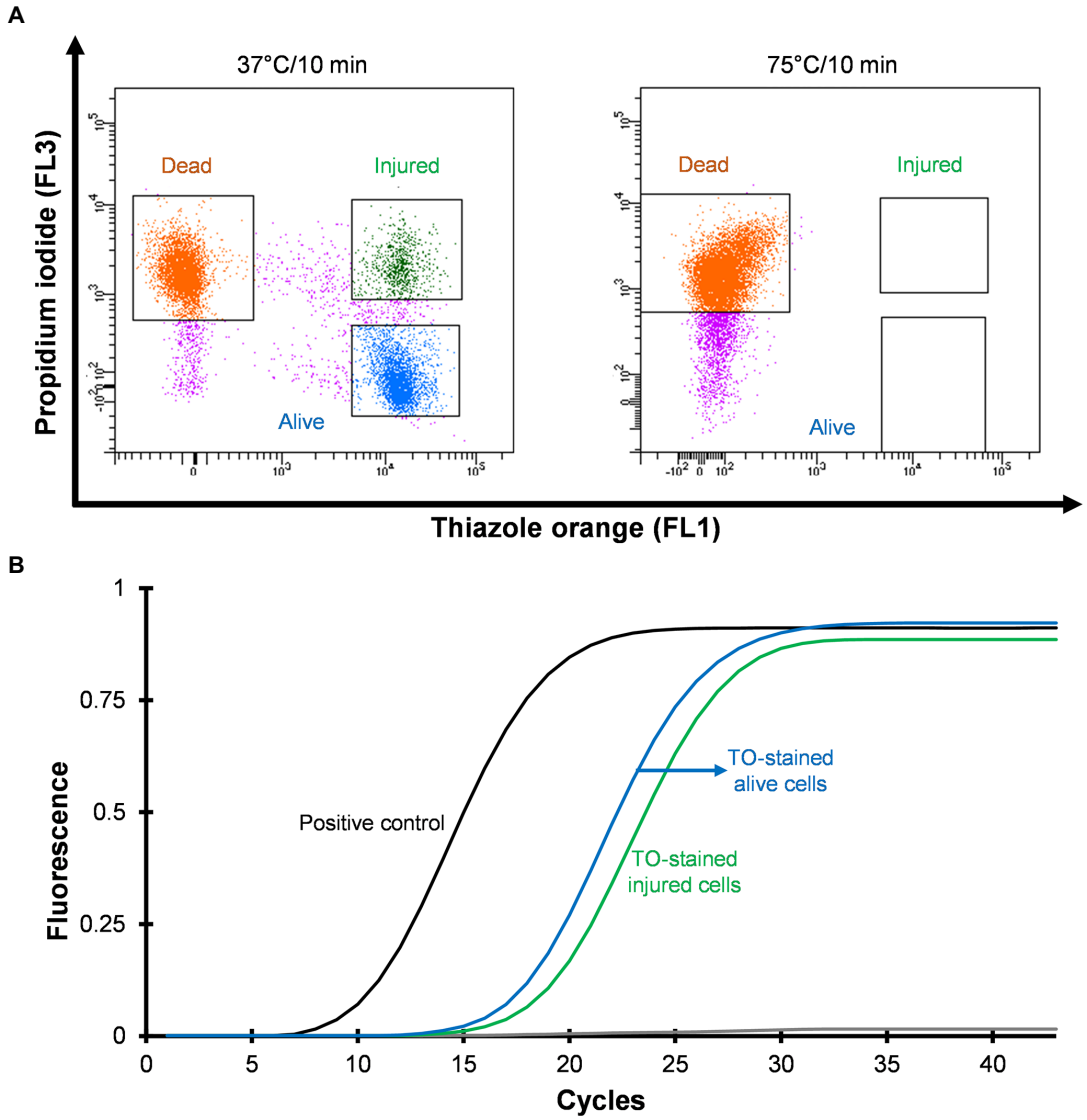
Viability based flow cytometry-cell sorting and qPCR assay. *L. pneumophila* grown on BCYE-GVPC agar at 37°C were used as a positive control and cells treated at 75°C for 10 min were used as a negative control. **(A)** Cytograms represent three populations: thiazole orange-stained alive (blue), thiazole orange-stained injured (green) and propidium iodide-stained dead (orange) cells. Propidium iodide selectively stained dead cells. Pink colored events are debris. **(B)** qPCR assay of thiazole orange stained alive and injured cells. The results showed that thiazole orange (TO) does not interfere with DNA extraction and qPCR.

Three distinct regions can be distinguished on the cytograms: PI-stained dead cells, TO-stained alive cells and TO-stained injured (transition state) cells (Figure 2A). Injured or intermediate cells were observed in between the alive and dead cell regions. The principle of the PI/TO assay is to selectively stain dead bacteria (damaged membrane) with the dye PI, whereas TO can enter into all cells (Alsharif and Godfrey, 2002). The PI staining divides cells into two major populations, i.e., PI-staining (dead) and PI-unstained (alive and injured) cells. In PI unstained cell population, fluorescence of TO dye become very prominent. Injured cells are in a transitional physiological state and stain positive with TO dye. In these experiments *Legionella* was grown at 37°C on agar for 3–4 days and all samples consisted of some dead, injured, and alive cells although the proportion of alive cells was the highest. It is possible that the washing, and centrifugation steps could have generated these small number of injured and dead cells. Different handling and processing steps have been shown to cause *Legionella* to lose culturability (Whiley, 2016). Figure 2A shows that thermal treatment at 75°C for 10 min completely inactivated the cells and these dead cells are stained with PI. These results clear demonstrated that using flow cytometry-cell sorting, it was possible to isolate and characterize all three cell fractions.

#### 3.2.2. Validation of qPCR and detection of VBNC *Legionella*

Mean C_T_ values obtained from qPCR assays and logarithmic concentration of 10-fold dilutions of *Legionella* 16S rDNA gBlocks were used to plot a standard curve with a 0.9995 regression coefficient and 88.9912% amplification efficiency. In this study, the limit of detection and limit of quantification were 5 GU and 25 GU, respectively. To investigate the effect of the TO dye, 10^3^–10^5^ sorted *Legionella* cells were used for DNA extraction. The extracted DNA was subjected to qPCR assay. Figure 2B illustrates that the TO dye did not interfere with DNA extraction and amplification.

### 3.3. Assay validation using pure cultures of *Legionella*

#### 3.3.1. Heat treatment promoted formation of VBNC *Legionella*

**Figure 3:**
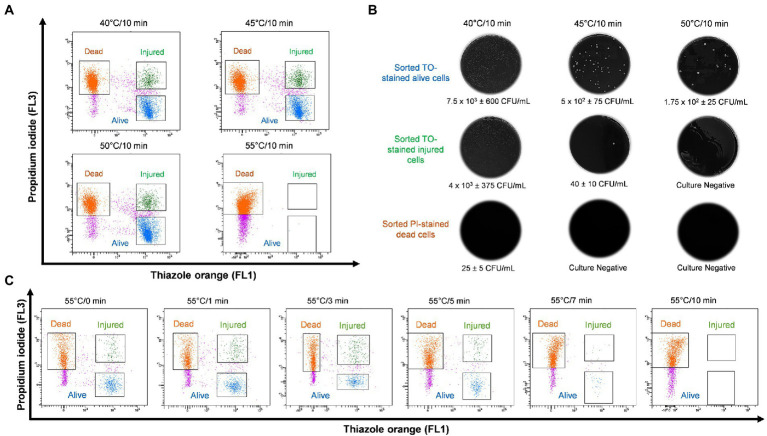
*Legionella pneumophila* thermal profile and culturability assay. **(A)** Bacterial cells were heat shocked at 40–55°C for 10 min and analyzed using flow cytometry. **(B)** Sorted cell fractions cultured on BCYE agar. Thermal shock at 50°C for 10 min transformed TO-stained injured cells into VBNC *Legionella*, as indicated by the lack of growth on the BCYE agar. **(C)** Pure culture of *L. pneumophila* subjected to 55°C thermal treatment in increments from 0 to 10 min. The population of VBNC *L. pneumophila* increased gradually with time 9% at 1 min, 20% at 3 min and then decreased to 5% at 5 min.

In order to study thermal tolerance of *Legionella*, bacteria were heat shocked sequentially in 5°C increments from 40 to 70°C for 10 min and examined by flow cytometry (Figure 3A). From the cytograms, PI-stained cells were non-culturable and non-viable *Legionella* and defined as dead cells. TO-stained alive cells grew on culture medium, whereas TO-stained injured cells obtained from ≤45°C treatment was unable to grow on both BCYE agar and *Legionella* enrichment broth (Figure 3B). The 50°C treatment triggered the formation of non-culturable injured cells, which were considered VBNC *Legionella*. Heat treatment at 55°C for 0–3 min increased the rate of transformation of alive cells into VBNC cells (Figure 3C). It is worth noting that a considerable number of *Legionella* converted into VBNC cells after 3 min of treatment. During the 55°C thermal treatment for 0–10 min, the number of PI-stained dead cells increased proportionally with the duration of thermal treatment. After 10 min of heat shock treatment at 55°C, all bacteria clustered in the dead cell region on the cytogram and were no longer culturable on BCYE agar or in *Legionella* enrichment broth. Thus, according to the cytograms (Figures 3A,C), the PI/TO membrane integrity assay is an effective way to study the effect of disinfection treatment on *Legionella*.

#### 3.3.2. Culturability and viability of VBNC *Legionella*

**Figure 4:**
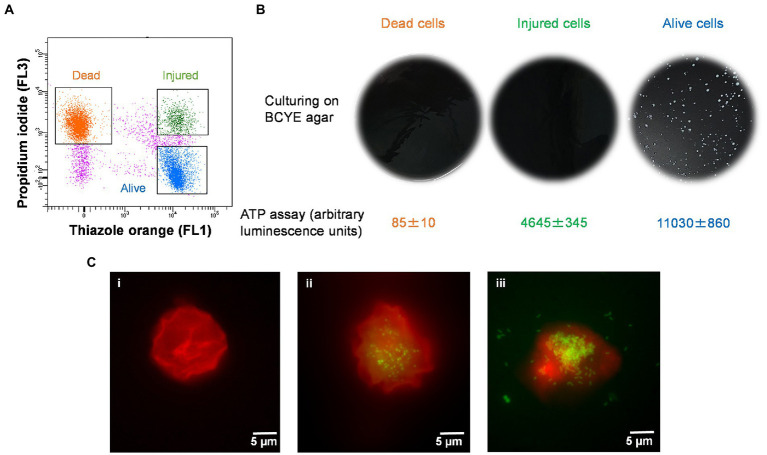
Characterization of thermally generated (50°C/10 min) VBNC *L. pneumophila*. **(A)** Cytogram representing thiazole orange-stained alive (blue), thiazole orange-stained injured (green) and propidium iodide-stained dead (orange) cells generated by 50°C heat shock for 10 min. **(B)** These three fractions were cultured on BCYE agar, and their ATP contents measured using a luciferase-based ATP estimation assay. **(C)**: FISH of *A*. *polyphaga* with Alexa Fluor 647 labeled EUK1209 (red) and *Legionella* with Alexa Fluor 488 labeled LEG705 (green) probes. **(Ci)** uninfected *A*. *polyphaga*. **(Cii,iii)** These micrographs show intracellular proliferation of VBNC *Legionella* within *A*. *polyphaga* after 7 and 10 days of infection, respectively.

The injured cells isolated following 50°C heat shock was unable to grow on either agar or in broth medium (Figures 4A,B). The viability of these cells was confirmed by quantification of ATP contents and resuscitation in *A. polyphaga*. According to the luciferase-based ATP detection assay, TO-stained alive culturable cells produced the highest amount of ATP (11,030 ± 860 units), whereas the least amount of ATP was detected from PI-stained dead cells (85 ± 10 units). TO-stained injured non-culturable cells were found to be metabolically active and able to produce 4,645 ± 345 units ATP.

Axenic *A. polyphaga* cultures were used to study infectivity of suspected VBNC (TO-stained injured) and culturable *Legionella*. This was achieved by labeling *Legionella* infected *A. polyphaga* cells with fluorescent probes and examining them under a fluorescent microscope. In the FISH images (Figure 4C), the LEG705 *Legionella* probe appears green and the EUK1209 eukaryotic probe is colored red. *A. polyphaga* appeared as red colored spherical bodies (Figure 4C), whereas *Legionella* appears as distinct cells localized within the amoebae cytoplasm (Figures 4Cii,iii). Unlike the VBNC *Legionella*, culturable *Legionella* were able to proliferate within *A. polyphaga* after 60 h of infection. The results from 7 days post-infection showed internalization and multiplication of suspected VBNC *Legionella* in *A. polyphaga* (Figure 4Cii). After 10 days of infection, host amoebae lost its cellular integrity and *Legionella* egressed (Figure 4Ciii). This demonstrates that thermally generated non-culturable injured cells exhibit intracellular proliferation within *A. polyphaga*. After 120 h of infection, the alive *Legionella* infected a higher number of *A. polyphaga* ≈ 23%, whereas the VBNC *Legionella* infected only ≈ 7% of *A. polyphaga*. Unlike alive and VBNC *Legionella* cells, the thermally inactivated *Legionella* (75°C for 10 min) were unable to proliferate within *A. polyphaga* cells. Furthermore, the differences with proliferation and infection rates suggest that cellular behavior and pathogenicity of culturable and VBNC *Legionella* is not identical. Both assays clearly establish that TO-stained injured non-culturable cells are metabolically active and infect *A. polyphaga* and can be regarded as VBNC *Legionella*.

### 3.4. Detection and quantification of VBNC from *Legionella* spiked water samples

**Figure 5:**
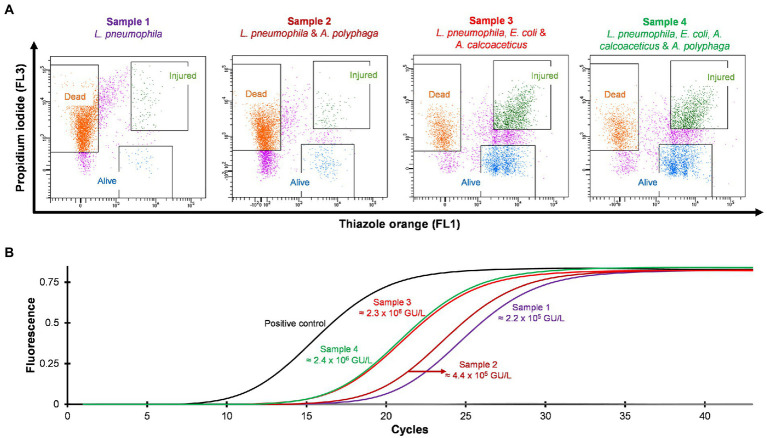
Detection and quantification of VBNC *L. pneumophila* from spiked water samples. All four spiked water samples were heated at 50°C for 15 min to generate VBNC *Legionella*. **(A)** Cytograms of four different water samples spiked with 4.5 × 10^6^ CFU/L *L. pneumophila*. Samples 3 and 4 generated the highest amount of VBNC cells. **(B)** qPCR of VBNC (collected injured fraction) *Legionella* taken from the four samples.

Four different water samples spiked with *L. pneumophila* were prepared and heat treated at 50°C for 15 min. Standard culturing method confirmed that all samples were negative for culturable *Legionella*. Using the flow cytometry-cell sorting assay these water samples were examined. It was observed that water samples (sample 3 and 4) consisted of mixed bacterial population generated a large population of TO-stained injured cells and a smaller population of PI-stained dead cells (Figure 5A). The qPCR results of injured cell fractions showed that water samples containing mixed bacterial populations harbored the highest amount of TO-stained injured *Legionella* cells (potentially VBNC cells ≈2 × 10^6^ GU/L, Figure 5B). It demonstrated that heat treatment of *Legionella* spiked water samples resulted in generation of VBNC *Legionella* and mixed bacterial cultures significantly increased the numbers of VBNC *Legionella*. This could potentially be due to the additional mixed bacteria cells providing a slight protection to the *Legionella* during the heat treatment or the mixed bacterial population harbored more biomass which increased the efficiency of DNA extraction and artificially increased the counts of *Legionella*. More importantly, in combination the PI/TO staining flow cytometry-cell sorting and qPCR assays can detect and quantify VBNC *Legionella* from potable water.

### 3.5. Detection and quantification of VBNC *Legionella* from environmental samples

**Figure 6:**
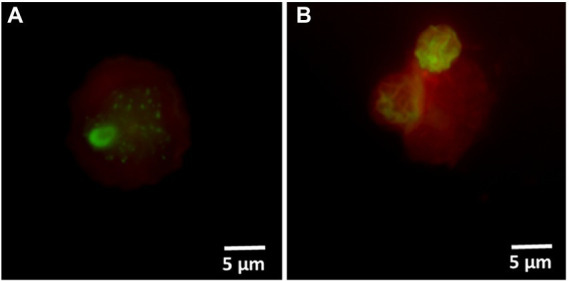
FISH of *A*. *polyphaga* stained with Alexa Fluor 647 labeled EUK1209 (red) and *Legionella* stained with Alexa Fluor 488 labeled LEG705 (green) probes. These micrographs show intracellular proliferation of environmental (shower water sample 1: **A** and sample 2: **B**) non-culturable *Legionella* within *A*. *polyphaga*.

**Figure 7:**
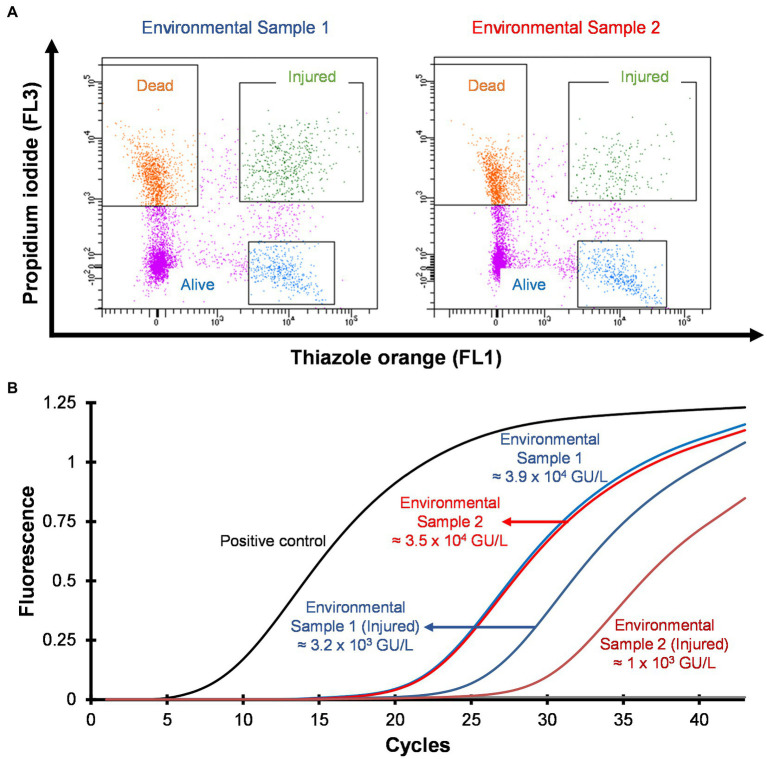
Detection and quantification of VBNC *L. pneumophila* from potable water samples. **(A)** Cytograms of culture negative potable water samples. **(B)** qPCR of whole water sample and VBNC *Legionella* and estimation of GU/L after viability cell sorting using flow cytometry shown in **(A)**. This image clearly demonstrates the difference in the concentration of *Legionella*, indicating presence of dead *Legionella* of its DNA in the water sample.

Hospital shower water samples were screened for *Legionella* using the Legiolert kit, standard *Legionella* culturing and qPCR assays. The two samples were selected that were negative for culturable *Legionella*, but the qPCR assay demonstrated the presence of *Legionella* DNA (Supplementary Table S1 shows the concentrations of the different *Legionella* populations enumerated using each method). Furthermore, the amoebae coculture assay and FISH analysis confirmed that non-culturable *Legionella* present in both samples effectively propagate in *A. polyphaga* after 7 days of infection (≈2% infected cells; Figure 6). To test efficacy of the VFC + qPCR assay both samples were stained with PI/TO dyes and analyzed by flow cytometry and injured cell fractions were sorted out. Figure 7A shows that both samples contained a significant amount of TO-stained injured cells, with environmental sample 1 having 1.47 × 10^5^ TO-stained injured cells and sample 2 contained 7.4 × 10^4^ TO-stained injured cells. DNA was extracted from the whole water sample and TO-stained injured cells and subjected to qPCR assay for the estimation of *Legionella* GU/L. Figure 7B clearly shows the difference between *Legionella* GU/L of the whole water sample and TO-stained injured cells. Environmental sample 1 contained 3.9 × 10^4^ GU/L *Legionella* whereas in TO-stained injured cells it was 3.2 × 10^3^ GU/L. Similarly, in sample 2, the *Legionella* concentration was 3.5 × 10^4^ GU/L in the whole sample whereas TO-stained injured cells contained 1 × 10^3^ GU/L *Legionella*. The difference (1 to 1.5 Log_10_) between *Legionella* genomic load of whole potable water sample and TO-stained injured cells indicated presence of dead *Legionella* or its residual DNA. These results challenge the efficacy and reliability of the culturing (ISO11731:2017-05) and standard qPCR assays (ISO/TS12869:2019).

## 4. Discussion

VBNC state is a physiological condition in which *Legionella* lose its ability to grow on standard microbiological culture media but retain cellular viability and pathogenicity (Epalle et al., 2015). It is unclear whether the VBNC state is an adaptive response supporting survival and persistence of *Legionella* under unfavorable conditions or a form of cellular damage, which results in loss of culturability. This presents an important challenge in *Legionella* research, as it is vital to understand the role of VBNC *Legionella* within manufactured water systems to improve control and risk management strategies to prevent LD. One cause of long-term persistence of *Legionella* contamination in engineered water systems is regular reseeding of *Legionella* and VBNC *Legionella* from the supply/source water (Whiley et al., 2017; Nisar et al., 2020). Common building water disinfection procedures including pasteurization, chlorination, and chlorine dioxide treatment, induces and selects VBNC *Legionella* (Allegra et al., 2008; Mustapha et al., 2015; Whiley et al., 2017). Importantly, in hospital buildings thermal disinfection can act as a selection pressure that results in the persistence of heat resistant *Legionella* (Allegra et al., 2011). The method described in this study successfully detected VBNC *Legionella* from environmental water samples (hospital showers). The viability-based flow cytometry assay clearly discriminated *Legionella* into dead and viable fractions (Figure 2A). The injured cells sorted in this assay, met the definition of VBNC *Legionella* as they were unculturable on standard BCYE agar (Figure 4B); however, were still viable as determined by ATP quantification and the ability to infect amoebae hosts (Figure 4C). These are accepted methods to measure viability, with ATP estimation kits widely used to discriminate metabolically active (alive or injured) cells from metabolically inactive (dead) cells (Allegra et al., 2008). Amoebae coculture assay is described as most suitable system to determine pathogenicity of VBNC *Legionella* (La Scola et al., 2001; Cervero-Arago et al., 2019). According to these results, the viability-based flow cytometry assay is an effective parameter to study behavior and physiology of *Legionella* under different disinfection treatments.

Complete eradication of *Legionella* from manufactured water systems is not practically possible; therefore, environmental monitoring and risk assessment of *Legionella* on a regular basis are crucial to reduce the risk of legionellosis outbreaks. Despite recent advances in molecular biology and biotechnology, the culture based approach is regarded as the standard method to detect *Legionella* contamination in engineered water systems (International Organization for Standardization, 2017; Standards Australia, 2017). Importantly in engineered water systems, the majority of the *Legionella* population exists in the VBNC state (Diederen et al., 2007). Routine detection and quantification methods, culture based or qPCR assay, either over or underestimate *Legionella* contamination and do not provide information about VBNC *Legionella* (Whiley, 2016).

There are previously described alive/dead dye-based flow cytometry protocols used to differentiate between alive and VBNC *Legionella* (Allegra et al., 2008; Zacharias et al., 2015; Braun et al., 2019). However, these previous protocols are designed to study pure cultures of *Legionella* and are not designed for analyzing environmental samples in which *Legionella* are present in complex microbial communities. When these previously described methods are applied to environmental samples they can differentiate the dead, VBNC, and alive total bacterial populations, but they cannot determine if *Legionella* is part of these populations (Li et al., 2014; Ramamurthy et al., 2014). One approach that has been previously used to overcome this limitation is the use of specific fluorogenic antibodies; however, this approach is currently strain specific and cannot be used to detect different environmental strains of *Legionella* (Füchslin et al., 2010). In contrast, the method described in this paper (VFC + qPCR) can detect and quantify VBNC *Legionella* from environmental samples. It combines alive/dead dye-based flow cytometry with cell sorting of the separate dead, VBNC and alive bacterial populations followed by qPCR enumeration of *Legionella* spp. and *L. pneumophila* present in the VBNC and alive populations. This study used PI and TO dyes to differentiate the alive, dead and VBNC bacterial populations. The PI selectively stains dead cells with damaged cell membranes (Crowley et al., 2016). The TO dye is then used to characterize the cells which do not uptake the PI (Alsharif and Godfrey, 2002; Allegra et al., 2008). A limitation of this approach is that PI has been shown to have reduce penetration of biofilm associated bacterial cells, which can lead to and overestimation of bacterial viability (Rosenberg et al., 2019).

This study explored the potential of thermal disinfection to induce the VBNC state. The findings demonstrated that culturability on the standard medium (BCYE and BCYE-GVPC agar) was the least reliable cellular viability indicator (Figures 3B, 4B). In addition, the effect of the acid and heat pre-treated methods (prescribed in the ISO11731:2017-05 culture method) on *Legionella* recovery was examined. These pre-treatments are included in the standard culture method to selectively kill other environmental bacteria and prevent plates from becoming overgrown (International Organization for Standardization, 2017). However, this study (Figure 1), demonstrated that both selective decontamination methods statistically significantly reduced the recovery of culturable *Legionella* and analysis using this described method showed that this reduction was caused by the induction of the VBNC state. This could potentially explain some of the unreliability and lack of reproducibility often observed with the ISO11731:2017-05 method (De Luca et al., 1999; Lucas et al., 2011). Other factors which compromise the validity of culture-based techniques are sample holding time (McCoy et al., 2012) and residual disinfectant (Wiedenmann et al., 2001) present in collected water sample. The limit of detection for this described method (VFC + qPCR) when applied to the environmental water samples was 10^2^ GU/L. This is comparable to the limit of detections for the standard ISO methods (10^2^ CFU/L for ISO11731:2017-05 and 10^2^ GU/L for ISO/TS12869:2019). Supplementary Figure S3 presents a comparison of *Legionella* CFU/L and GU/L and shows they were comparable at concentrations from 10^3^ to 10^8^ CFU/L or GU/L.

In conclusion, this study describes a direct and rapid (results within 5–6 h) assay to detect and quantify VBNC *Legionella* from potable water samples. This assay offers following advantages: (a) viable and dead *Legionella* quantification without culturing (b) quantification of viable and dead *Legionella*; and (c) rapid and direct screening of VBNC *Legionella*.

## Data availability statement

The original contributions presented in the study are included in the article/Supplementary material, further inquiries can be directed to the corresponding author.

## Author contributions

MN, HW, and RB conceived and designed the research. MN performed the experiments. GB and MN conducted flow cytometry-cell sorting assay. KR, MB, HW, and RB provided technical assistance. MN and HW drafted and edited the manuscript. HW, KR, MB, GB, and RB corrected and contributed to the manuscript. All authors contributed to the article and approved the submitted version.

## Conflict of interest

The authors declare that the research was conducted in the absence of any commercial or financial relationships that could be construed as a potential conflict of interest.

## Publisher’s note

All claims expressed in this article are solely those of the authors and do not necessarily represent those of their affiliated organizations, or those of the publisher, the editors and the reviewers. Any product that may be evaluated in this article, or claim that may be made by its manufacturer, is not guaranteed or endorsed by the publisher.
