# Supplementary material for: Detection and quantification of viable but non-culturable Legionella pneumophila from water samples using flow cytometry-cell sorting and quantitative PCR
Source: Front Microbiol. 2023 Jan 30;14:1094877. doi: 10.3389/fmicb.2023.1094877 (PMC9922708; doi:10.3389/fmicb.2023.1094877)
Supplement: Supplementary file 1 [file Data_Sheet_1.docx]

Detection and quantification of viable but non-culturable (VBNC) *Legionella pneumophila* from water samples using flow cytometry-cell sorting and quantitative PCR

Muhammad Atif Nisar^1^, Kirstin E Ross^1^, Melissa H Brown^1^, Richard Bentham^1^, Giles Best^2,3^, Harriet Whiley^1^

^1^College of Science and Engineering, Flinders University, Bedford Park, SA, 5042 Australia

^2^College of Medicine and Public Health, Flinders University, Bedford Park, SA 5042, Australia

^3^Flow Cytometry Facility, Flinders University, Bedford Park, SA 5042, Australia

***Correspondence:**Corresponding Author

Harriet.Whiley@flinders.edu.au

Supplementary Material

# Supplementary Figures and Tables

**Figure S1:** Cytogram represent “staining buffer” stained with “thiazole orange” and “propidium iodide” dyes.

**Figure S2:** Flow cytometry of *L. pneumophila* grown on BCYE-GVPC agar at 37°C. A: Discrimination of bacterial population based on forward (FSC) and side scatter (SSC) parameters. B and C: Cytograms represent three different bacterial populations: thiazole orange-stained alive (blue), thiazole orange-stained injured (green) and propidium iodide-stained dead (orange) cells.

**Figure S3:** Relationship between *L. pneumophila* genomic unit (GU/L) enumerated using qPCR and colony forming units (CFU/L) enumerated using culture. Six different dilutions of pure *L. pneumophila* culture (concentrations ranging from 10^3^ to 10^8^ CFU/L) were processed according to ISO/TS12869:2019 and ISO11731:2017-05.

**Table S1:** Comparison of Legiolert kit, standard *Legionella* culturing and qPCR assays, amoebae co-culture assay and “viability based flow cytometry-cell sorting and qPCR” assay on two environmental samples.

## Supplementary Figures


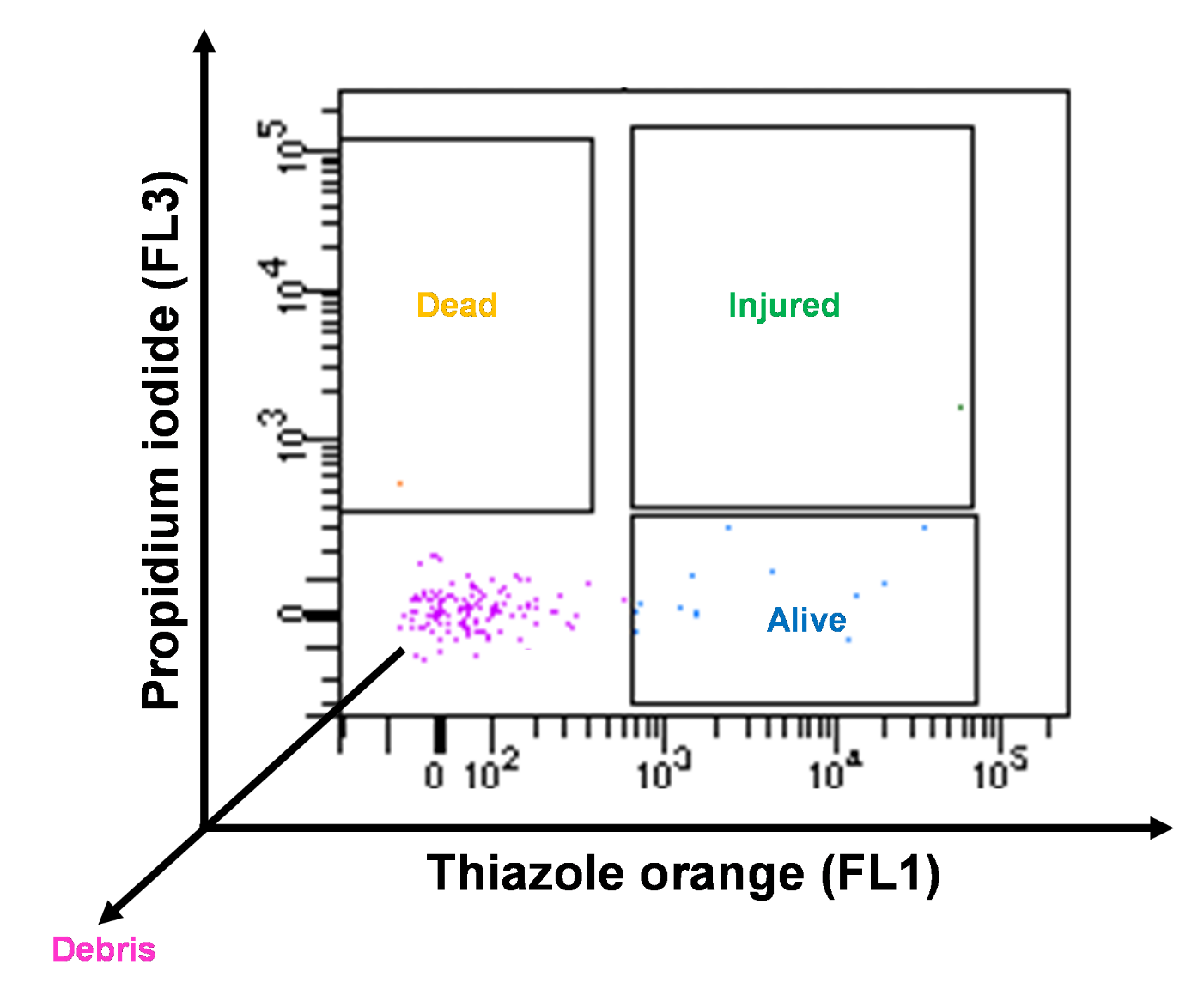


**Figure S1:** Cytogram represent “staining buffer” stained with “thiazole orange” and “propidium iodide” dyes.


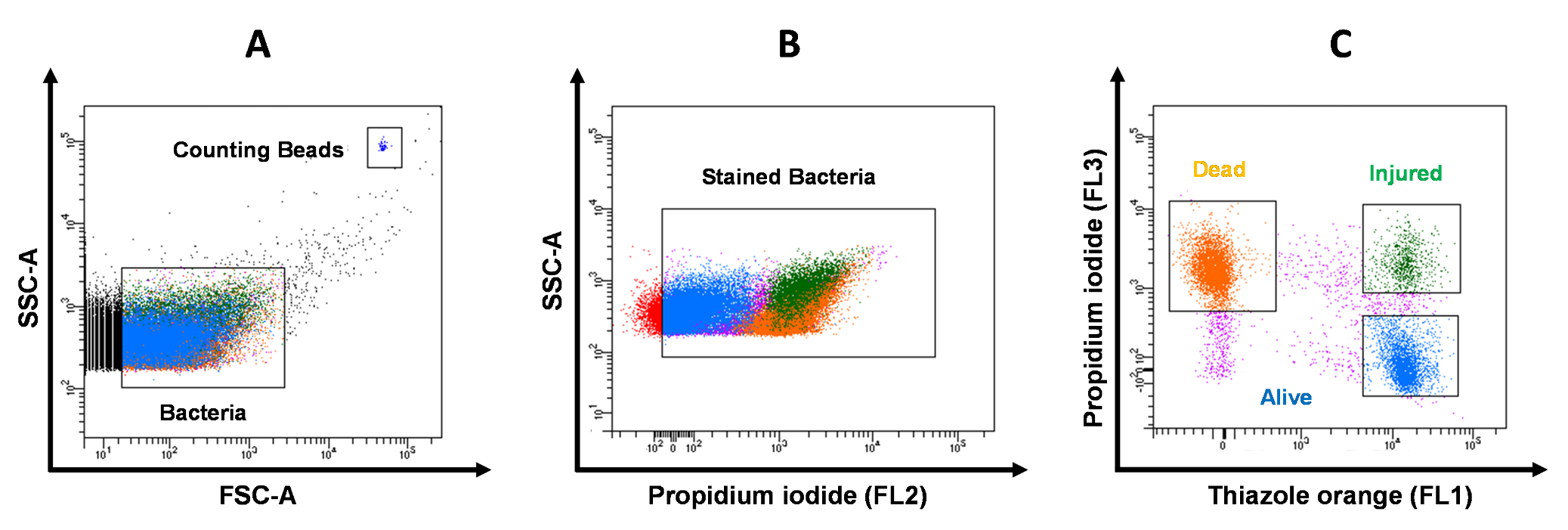


**Figure S2:** Flow cytometry of *L. pneumophila* grown on BCYE-GVPC agar at 37°C. A: Discrimination of bacterial population based on forward (FSC) and side scatter (SSC) parameters. B and C: Cytograms represent three different bacterial populations: thiazole orange-stained alive (blue), thiazole orange-stained injured (green) and propidium iodide-stained dead (orange) cells.


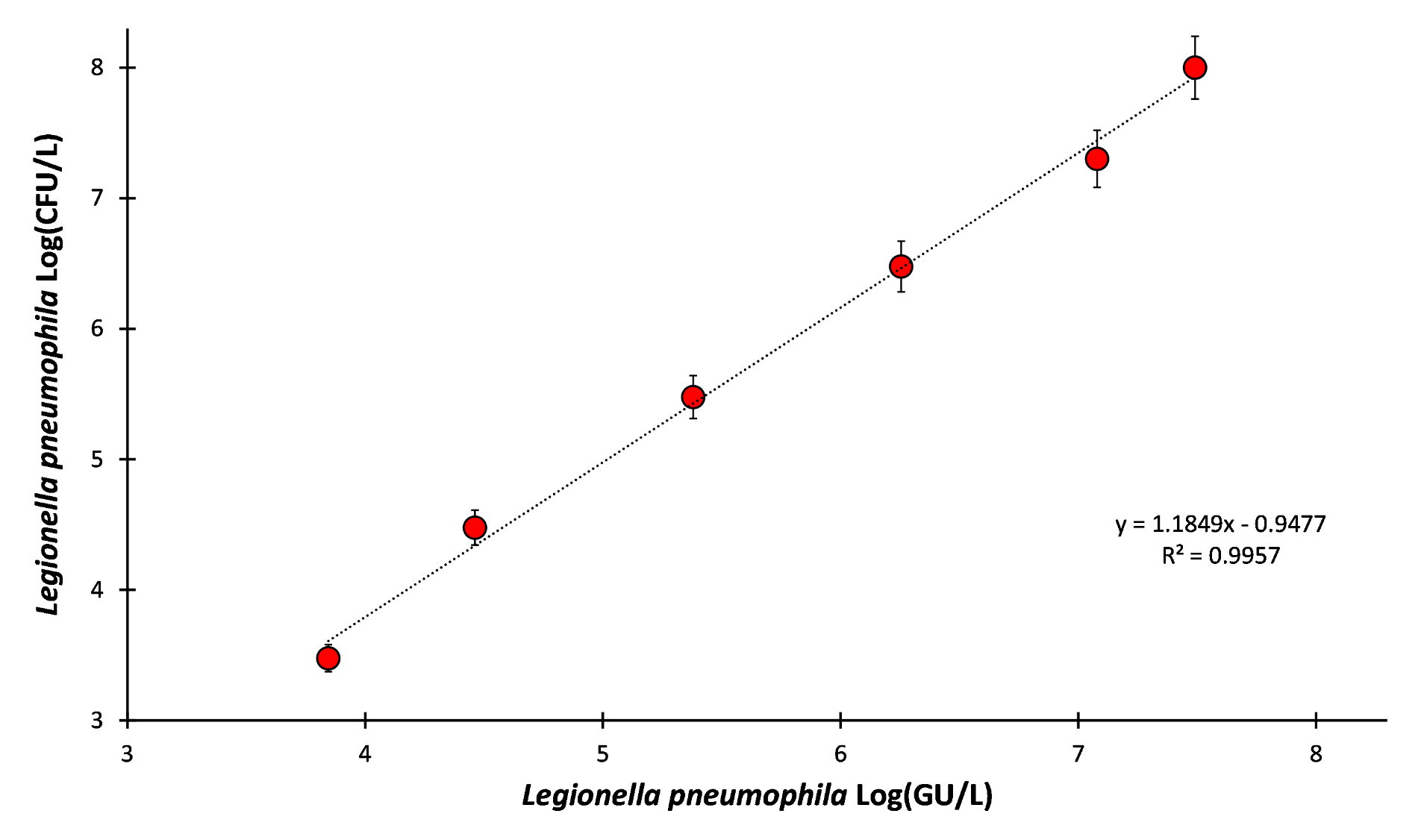


**Figure S3:** Relationship between *L. pneumophila* genomic unit (GU/L) enumerated using qPCR and colony forming units (CFU/L) enumerated using culture. Six different dilutions of pure *L. pneumophila* culture (concentrations ranging from 10^3^ to 10^8^ CFU/L) were processed according to ISO/TS12869:2019 and ISO11731:2017-05.

## Supplementary Table

**Table S1:** Comparison of Legiolert kit, standard *Legionella* culturing and qPCR assays, amoebae co-culture assay and “viability based flow cytometry-cell sorting and qPCR” assay on two environmental samples.

| Sample | Standard Culturing | Legiolert kit | Amoebae co-culture assay | Standard qPCR assay | Viability based flow cytometry-cell sorting and qPCR assay |
| --- | --- | --- | --- | --- | --- |
| Shower water 1 | Negative | Negative | Positive | 3.9 x 10^4^ GU/L | 3.2 x 10^3^ GU/L |
| Shower water 2 | Negative | Negative | Positive | 3.5 x 10^4^ GU/L | 1 x 10^3^ GU/L |
